# Neurofeedback Training for Opiate Addiction: Improvement of Mental Health and Craving

**DOI:** 10.1007/s10484-013-9218-5

**Published:** 2013-04-20

**Authors:** Fateme Dehghani-Arani, Reza Rostami, Hosein Nadali

**Affiliations:** 1 Tehran, Islamic Republic of Iran; 2 Department of Psychology, University of Tehran, Tehran, 1969713663 Iran

**Keywords:** Neurofeedback, Opiate addiction, Mental health, Craving

## Abstract

Psychological improvements in patients with substance use disorders have been reported after neurofeedback treatment. However, neurofeedback has not been commonly accepted as a treatment for substance dependence. This study was carried out to examine the effectiveness of this therapeutic method for opiate dependence disorder. The specific aim was to investigate whether treatment leads to any changes in mental health and substance craving. In this experimental study with a pre-post test design, 20 opiate dependent patients undergoing Methadone or Buprenorphine maintenance treatment were examined and matched and randomized into two groups. While both experimental and control groups received their usual maintenance treatment, the experimental group received 30 sessions of neurofeedback treatment in addition. The neurofeedback treatment consisted of sensory motor rhythm training on Cz, followed by an alpha-theta protocol on Pz. Data from the general health questionnaire and a heroin craving questionnaire were collected before and after treatment. Multivariate analysis of covariance showed that the experimental group achieved improvement in somatic symptoms, depression, and total score in general mental health; and in anticipation of positive outcome, desire to use opioid, and relief from withdrawal of craving in comparison with the control group. The study supports the effectiveness of neurofeedback training as a therapeutic method in opiate dependence disorder, in supplement to pharmacotherapy.

## Introduction

Substance use disorder is characterized by physiological dependence accompanied by the withdrawal syndrome upon abstinence from the drug, psychological dependence with craving, a pathological motivational state that leads to active drug-seeking behavior, and tolerance, expressed in the escalation of the dose needed to achieve a desired euphoric state (Sadock and Sadock 2008). It is a chronic, relapsing mental disease that results from the prolonged effects of drugs on the brain (Dackis and O’Brien 2001; Volkow et al. 2003, 2004). Opiate dependence refers to a cluster of substance use disorders with physiological, behavioral, and cognitive symptoms, which, taken together, indicate repeated and continuing use of opiate drugs despite significant problems related to such use (Sadock and Sadock 2008). Drug and opiate substance dependence can take control of the brain and behavior by activating and reinforcing behavioral patterns that are excessively directed to compulsive drug use (Di Chiara 1999; Gerdeman et al. 2003).

The effects of pharmacological and behavioral treatment for substance dependence have been criticized as being limited (Fagan 1994). While major resources have been employed to study and treat substance dependence, there has been minimal improvement in success rates of treatment and the relapse rate is typically over 70 % (Higgins et al. 1995). Gossop et al. (2002) reported 60 % of heroin addicts relapsed 1 year following substance dependence treatment. Effective treatment for substance dependence will always require a combined biological, physiological and psychological approach. Few treatment programs address the neurological and physiological issues of substance dependence (Sokhadze et al. 2008).

In recent years, the psychological and neurophysiologic dimensions of substance dependence have attracted more scientific attention (National Institute on Drug Abuse 2000). Volkow et al. (1988) were the first to use positron emission tomography (PET) to study the effects of cocaine on the human brain. This study has played a central role in ascertaining the interactions between the brain, drug and behavior in humans. Studies have shown that some symptoms of substance and opiate dependence such as craving, impulsiveness, and psychological abnormalities, are connected to pathological neurophysiology (Kaufman 2000; Dackis and O’Brien 2001; Hubbard and Martin 2001; Volkow et al. 2003, 2004; Ardila et al. 1991). Quantitative electroencephalography (QEEG) as a kind of brain mapping technique can characterize some of these abnormalities (Newton et al. 2003). The spontaneous EEG activity of substance and opiate dependence patients is characterized by alterations mainly within the alpha, theta and beta bands (Alper et al. 1998; Sokhadze et al. 2008), which may be the result of prolonged substance use itself (Ardila et al. 1991; Marchesi et al. 1992; O’Mahony and Doherty 1996).

The limitations of pharmacotherapy and behavioral therapy, combined with knowledge from studies on nerophysiological abnormalities in substance dependence, underline the need for alternative and/or complementary therapies for these disorders, with long lasting effects and minimal side effects (Hubbard and Martin 2001). Neurofeedback appears to be a promising alternative due to effects such as reduced drug seeking symptoms, improved psychological and neurophysiological variables and longer periods of abstinence that have been reported in the literature after neurofeedback treatment (Peniston and Kulkosky 1989; Masterpasqua and Healey 2003; Scott et al. 2005v Sokhadze et al. 2008).

Neurofeedback as a branch of biofeedback technology, is an operant conditioning technique used to reinforce or inhibit specific forms of EEG activity (Scott et al. 2005v Kaiser and Othmer 2000). It is a therapeutic method designed to train the mind and body to act in a more optimal way in order to improve emotional, cognitive, physical, and behavioral experiences (Demos 2005). Today, based on the research in neuropathology, we can use this method to turn abnormal rhythms and frequencies (based on QEEG) into normal (or relatively normal) rhythms and frequencies, and following that, turn abnormal psychological states into normal ones (Gunkelman and Johnstone 2005). Neurofeedback has been used as a therapeutic method to treat different types of disorders, for example attention deficit hyperactivity disorder (Kropotov et al. 2007; Strehl et al. 2006; Rossiter 2004; Fuchs et al. 2003), epilepsy (Kotchoubey et al. 2001), depression (Putman 2001), anxiety and affective disorders (Hammond 2005; Vanathy et al. 1998), fibromyalgia (Muller et al. 2001), and obsessive compulsive disorder (Hammond 2003), and also to enhance attention and memory performance in healthy subjects (Wilson et al. 2006; Hanslmayr et al. 2005; Egner et al. 2002; Vernon et al. 2003). This technique also has been used as a therapeutic method for substance or alcohol dependent patients, and results have corroborated the efficiency of neurofeedback treatment on negative neuropsychological consequences of these disorders (Sokhadze et al. 2008; Scott et al. 2005; Frederick et al. 2005; Burkett et al. 2004; Masterpasqua and Healey 2003; Lawrence 2002; Kaiser et al. 1999; Peniston and Saxby 1995).

Alpha training was the first neurofeedback (EEG biofeedback) protocol that was employed in substance and alcohol dependence disorders. Research by Passini et al. (1977) has shown the effects of alpha neurofeedback training in reducing anxiety and improving aspects of personality in drug dependence patients. Goldberg and Hillier (1979) reported that their alpha conditioning program reduced drug use and increased self-control in 4 patients addicted to opioids. Afterward the treatment of addictive disorders by EEG biofeedback or neurofeedback was popularized by the work of Eugene Peniston (Peniston and Kulkosky 1989, 1991) and became popularly known as the Peniston Protocol. In Peniston’s (1989) neurofeedback study, ten alcoholic patients underwent approximately 40 alpha-theta brain wave training sessions. These patients had all failed previous hospital residential treatment programs. Eight of them remained generally abstinent at least three years after neurofeedback treatment (Peniston and Kulkosky 1989). This protocol also resulted in a decrease of 13 on the millon clinical multiaxial inventory scales (MCMI), including anxiety, whereas traditional treatment produced decreases of only two points on these scales (Peniston and Kulkosky 1991).

In 1992, Fahrion, Walters, Coyne and Allen replicated these results in a controlled case study. They also reported a decrease in stress-related, blood based beta endorphins and in substance craving in patients. In researches completed by Bodehnamer and Callaway (2004) and Burkett et al. (2004) on crack-cocaine abusers improvements in mental state, craving and neurological functions have been reported. In another experimental study, participants who received neurofeedback treatment (alpha-theta training) showed significant improvement in their mood and in their Minnesota multiphase personality inventory-2 (MMPI-2) scales (Raymond et al. 2005). Follow-up studies showed the constancy of treatment outcomes in alcohol or drug addicted clients who completed an alpha-theta neurofeedback training program (Kelley 1997; Bodenhamer-Davis and BeBeus 1995; Trudeau 2000).

In a more recent study Scott et al. (2005) gave mixed substance dependence patients feedback on their brain’s electrical activity in conjunction with conventional treatment. They reported that their treatment doubled the recovery rate for drug dependence. In addition to improving the success rate for recovery from use of drugs, the study documented significant improvements in the ability of the experimental group to focus their thinking and information processing. Moreover, the experimental subjects exhibited significant improvement in some relevant measures of psychological functioning. After only 45 days of treatment, nearly one-third of the control group had dropped out of treatment prematurely and left the residential facility, compared to only 6 % for the experimental group. Even thought all of the works presented thus far were conducted on adults, Trudeau (2005) also suggested that neurofeedback could be effective for helping adolescents with substance use disorders.

Despite all these promising results, neurofeedback treatment has not yet beenaccepted as a standard therapy for substance dependence disorders because there are only a few studies in this field, and most of them have been conducted on alcoholic patients. In this study we examined the effectiveness of the neurofeedback method combined with pharmacotherapy in opiate dependence. We believe this is the first study to examine the effects of neurofeedback treatment in addition to Methadone or Buprenorphine maintenance treatment (MMT/BMT) on improvement of comorbid abnormalities in opiate dependent patients. A comprehensive assessment was carried out on general psychological health and substance craving. This study aimed at answering (a) if neurofeedback treatment leads to an improvement in mental health and craving for opiates and (b) if the two experimental and control groups differ in mental health and craving variables. This paper compares results from both groups.

## Materials and Methods

### Participants

The participants were 20 men, aged 20–50 years, who had opiate dependence disorder according to DSM-IV-R criteria. The main substances on which they were dependent were opium, heroin, and/or crack heroin. The route of administration was smoking. None of participants were intravenous addicts. They had no additional anoxia, head trauma, stroke, encephalitis or HIV. Participants were recruited from an outpatient clinic for substance dependence disorders treatment. They had received at least 3 months of Methadone or Buprenorphine maintenance treatment (MMT/BMT) for substance dependence disorder. Just two participants (one in the experimental group and another one in the control group) had been receiving Buprenorphine maintenance treatment. For patients under Methadone maintenance treatment, Suboxone, that has also Naloxone as a part of substance dependence pharmacotherapy, was the formulation of Methadone that had been used. The prescribed Methadone was in liquid form. During the incoming phase a complete blood and urine test had been taken from all participants. None of them had any substance usage during the last 10 days.

After providing informed consent, all 20 participants were initially evaluated for general psychological health and opiate craving. The patients were then randomized into the experimental and control groups, with the constraint that the groups be matched regarding age (average of 30 years old), education, and general health scores. Table 1 shows key demographic information for the two groups. Both groups were under Methadone or Buprenorphine maintenance treatment for substance dependence disorder. The experimental group also received 30 sessions of neurofeedback in addition to their usual MMT/BMT.

**Table 1:** Demographic data for the experimental and control groups

Group	N	Age			Education (years)			Abstinence (month)		
		Mean	SD	Range	Mean	SD	Range	Mean	SD	Range
Experimental	10	30.3	7.01	21–45	14.5	1.8	12–16	3.2	1.93	1–6
Control	10	29.1	6.5	21–40	14	1.9	12–17	3.6	2	1–7
Total	20	29.7	6.64	21–45	14.25	1.86	12–17	3.2	1.9	1–7

Indeed, the patients in this study, was the same as those contained in our previous publication (Dehghani-Arani et al. 2010) and the treatment procedure as well. But this paper examines a different set of measures on the subjects.

### Experimental Procedure

The neurofeedback program for the experimental group lasted 2 months (30 50-min sessions). The control group patients were receiving their usual treatment without neurofeedback. The neurofeedback training protocols in every session focused on Sensory Motor Rhythm (SMR) training in the Cz (the central brain cortex) area (Scott et al. 2005) and alpha-theta in the Pz (the parietal brain cortex) area (Peniston and Kulkosky 1989), each lasting 20 min, using a Thought Technology Procomp2 system.

The brain’s electrical activity was displayed on a monitor in the form of an audio-visual exercise. The feedback informed patients of their success in making changes. The training was introduced as a computer game in which they could score points by using their brain waves. Participants were advised to be attentive to the feedback and to find the most successful mental strategy to get as many points as possible. No other specific instructions were given to them.

In SMR training protocol on the Cz area, the active electrode was placed at Cz with a left-ear reference (A1). The right earlobe was connected to circuit ground. In this program the reinforcement band was SMR (12–15 Hz) frequency band, and the suppressed frequency were delta (2–5 Hz), theta (5–8 Hz) and high beta (18–30 Hz), frequency bands. Thresholds were adjusted in a way that if the participant maintained the reinforcement band above the threshold for 80 % of the time during at least 0.5 s, and the suppressed band under the threshold for 20 % of the time, feedback was received. Whenever participants could maintain the reinforcement band’s above the threshold for 90 % of the time during two continuous trials, the threshold was changed automatically so that it was closer to the optimal threshold (Scott et al. 2005).

Feedback in the alpha-theta training protocol on the Pz area was in audio format only. In this protocol, the participants closed their eyes, and only listened to the sound being played to them. Three pathways connected with this protocol were related to the theta (5–8 Hz), alpha (8–12 Hz), and beta (15–18 Hz) frequency bands, with one additional pathway to control delta (2–5 Hz). The initial sessions were used to train patients to decrease alpha levels that were above 12 mV (peak to peak), while augmenting theta, until there was ‘‘crossover.’’ This was defined as the point at which the alpha amplitude dropped below the level of theta. Subsequent to achieving the first crossover, both alpha and theta frequencies were augmented and the delta frequency range was also inhibited. This was intended to discourage the sleep transition during low-arousal states.

Each alpha-theta session began with the subject sitting in a chair with eyes closed. The active electrode was placed at Pz with a left-ear reference (A1) and right-ear ground (A2). Two distinct tones were employed for alpha and theta reinforcement, with the higher pitched sound used to index the higher-frequency alpha band. At the start of each session, the therapist spent 3–5 min reading a script of guided imagery to the experimental subject that dealt with identified essential elements of maintaining abstinence. After the guided imagery, it was made clear to the subject that the objective of the training did not involve explicit rehearsal of the script during the neurofeedback. Subjects reporting previous meditative practices were asked not to use them during the training, because meditation has been observed to override alpha-theta reinforcement effects (Scott et al. 2005). Following the alpha-theta training, subjects were given the opportunity to process their experience. When it appeared that the subject’s delta activity started to elevate and that sleep might be occurring during training, subjects were told prior to their next session to move a limb if they heard the therapist say for example “left hand”. Subsequently, during sessions where delta was elevating toward no responsiveness levels, the feedback sounds were inhibited in order to discourage the sleep transition. (Peniston and Saxby 1995; Scott et al. 2005).

### Instruments

The 28-item form of the general health questionnaire (GHQ-28) and the 45-item form of the heroin craving questionnaire (HCQ-45) were used to obtain general psychological health and opiate craving information before and after treatment.

The general health questionnaire (GHQ) is a self-administered screening questionnaire designed to detect probable psychiatric disorder in primary care settings (Goldberg 1972). It is highly popular and widely used in research (e. g., Lobo et al. 1986; Gureje and Obikoya 1990; Schmitz et al. 1999). It was developed by Goldberg and Hiller in 1972 for diagnosing non psychotic mental disorders in health centers. This questionnaire is equipped with the proper questions to ascertain the severity of mental disorders (Robins and Brooks 1981). Benjamin et al. (1983) have emphasized use of the shorter 28-item version of this questionnaire in order to save on costs and time in important research projects, when studying the general status of mental health of patients. The 28 section form of this questionnaire, compiled by Goldberg and Hillier (1979), has four subscales: physical signs, anxiety and sleep disorders, social disorders, and severe depression subscales. A Total score is also obtained. Reliability coefficients have ranged from 0.78 to 0.95 in various studies (Furukawa et al. 2001; Goldberg 1972).

The heroin craving questionnaire includes 45 questions with a 7 level Likert scoring system (with some items reverse scored). Respondents indicate the degree to which they agree with each statement along a 7-point Likert-type scale ranging from “Strongly disagree” to “Strongly agree”. This instrument provides five main subscales, all of which were included in analysis: anticipation of positive outcome, relief from withdrawal, intention and plan to use substance, desire to use substance, and lack of control over use. Research supports the validity and reliability (0.69–0.93) of the subsections of this questionnaire in measuring the severity of craving in patients with heroin or other opiate dependence disorders (Heinz et al. 2006; Sayette et al. 2000).

The results obtained in the pre and post-treatment phases for the experimental and control groups were analyzed by the SPSS.16 tool.

## Results

In order to check the effect of the pre-treatment phase in an effort to find whether neurofeedback plus pharmacotherapy (MMT/BMT) is more effective than pharmacotherapy alone, the multivariate analysis of covariance (MANCOVA) was used. For this purpose the scores in post-treatment subscales of general health questionnaire and Heroin Craving Questionnaire as the dependent variables, the intervention (in two levels) as the independent variable and the score of pre-treatment indexes as the covariate variables, were used for analysis. After checking the hypothesis of linearity, homogeneity of regression lines, and homogeneity of variances, the effect of intervention with the dependent variables was examined.

### General Health Questionnaire

**Fig. 1:**
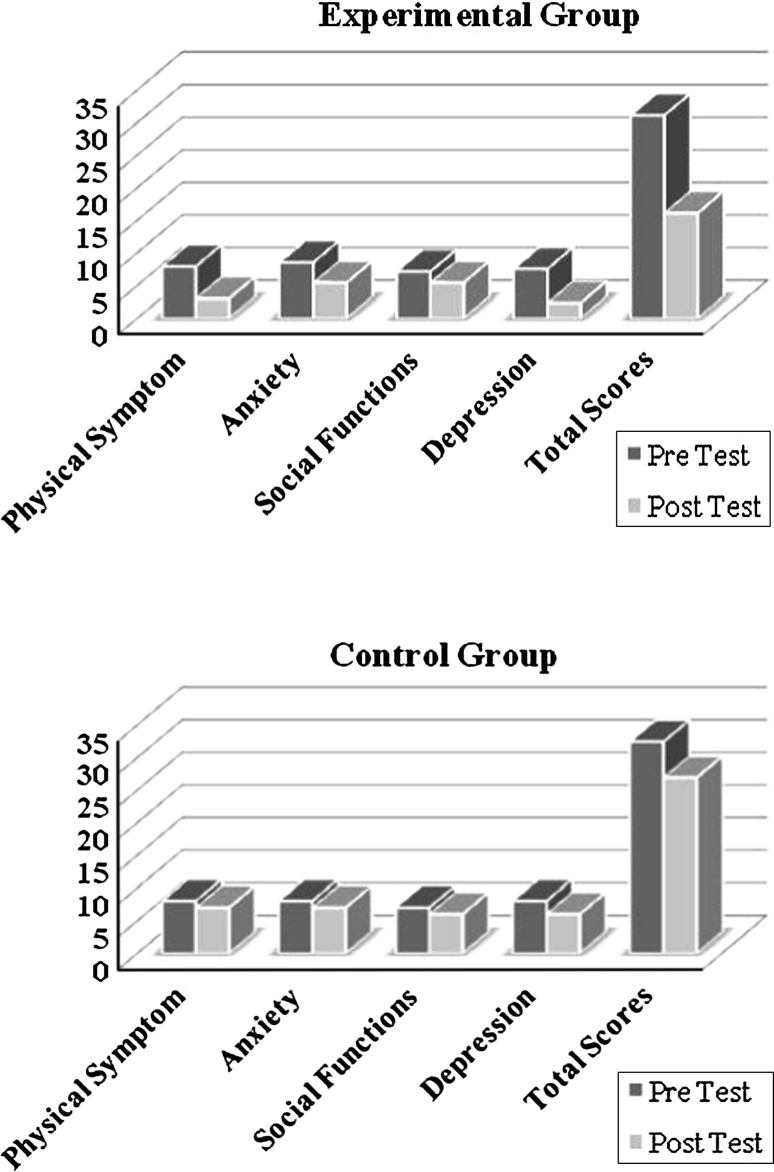
Pre and post results of GHQ subscales in Experimental and control groups

Descriptive statistics for the experimental and control groups, pre and post, for the GHQ-28 are shown in Table 2 and graphically displayed in Fig. 1. MANCOVA results are provided in Table 3, where it is seen that the intervention produced significant improvement for physical symptoms, depression, and the total score of mental health. It can be argued that the independent variable had caused a significant difference between the experimental and control groups. No differences were found for anxiety or social functions.

**Table 2:** Descriptive indexes for the GHQ-28 prior to and following treatment

Variables	Experimental				Control			
	Mean		Standard deviations		Mean		Standard deviations	
	Pre	Post	Pre	Post	Pre	Post	Pre	Post
Physical Symptom	7.9	3	3.6	2.44	8	7	3.6	4.16
Anxiety	8.5	5.4	3.13	2.71	8	7	3.57	4.9
Social Functions	7.1	5.4	4.53	2.75	7	6	4.88	3.4
Depression	7.5	2.3	5.7	2.4	8	6	6.24	5.08
Total Scores	31.1	16.1	11.64	8.03	32.4	26.9	15.32	11.68

**Table 3:** Results of MANCOVA for GHQ subscales in the experimental and control groups

Variable	F	Sig.	Eta squared
Physical symptom	6.37	.02*	.35
Anxiety	1.41	.25	.09
Social functions	.18	.67	.02
Depression	4.36	.04*	.27
Total scores	4.27	.04*	.26

df = (1,19)

* *p* < .05

### Heroin Craving Questionnaire

**Fig. 2:**
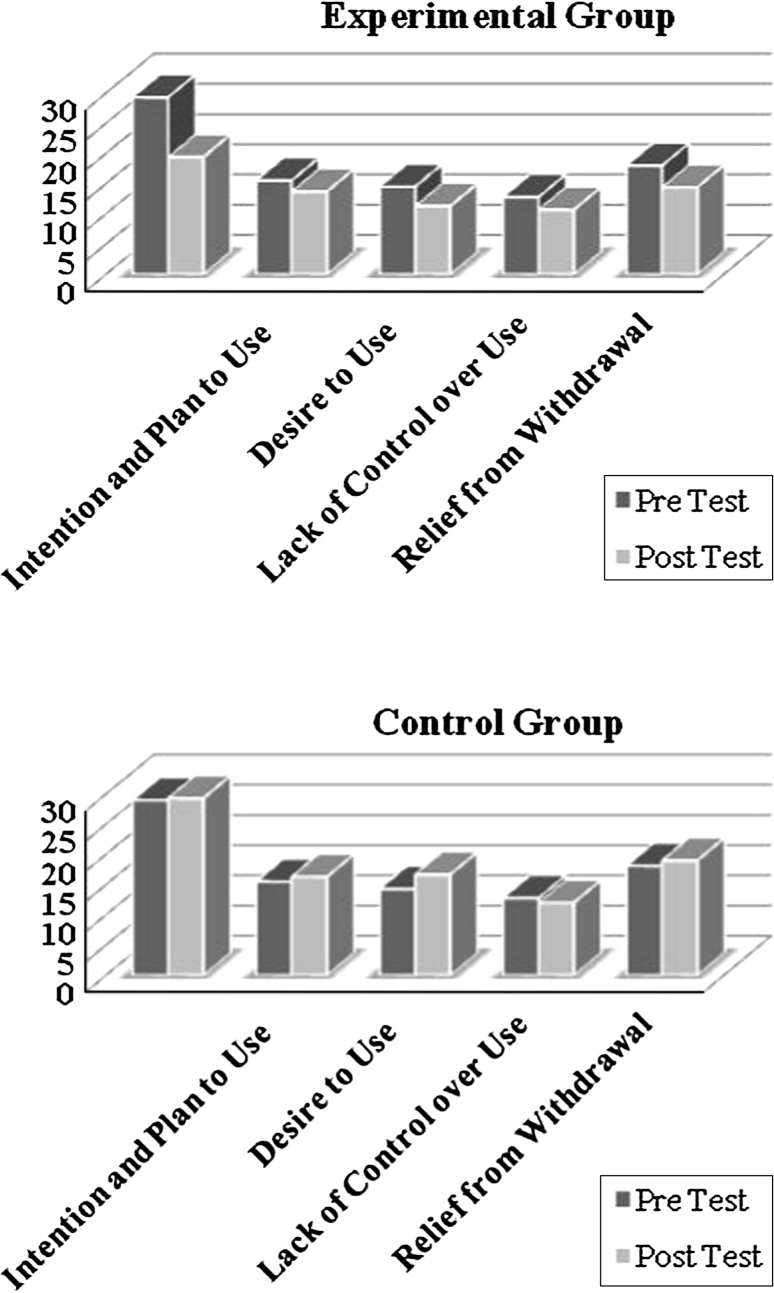
Pre and post results of HCQ subscales in Experimental and control groups

Descriptive statistics for the experimental and control groups, pre and post, for the HCQ may be found in Table 4 and graphically displayed in Fig. 2. MANCOVA results, provided in Table 5, show that the intervention led to significant improvements for anticipation of positive outcome, desire to use, and relief from withdrawal.No changes were noted for plan to use and lack of control.

**Table 4:** Descriptive indexes of the HCQ prior to and following treatment

Variables	Experimental				Control			
	Mean		Standard deviations		Mean		Standard deviations	
	Pre	Post	Pre	Post	Pre	Post	Pre	Post
Anticipation of positive outcome	29.3	19.4	8.65	4.11	29	29.3	8.56	15.82
Intention and plan to use	15.53	13.7	5.95	5.63	15.5	16.3	5.96	8.08
Desire to use	14.5	11.3	5.93	5.37	14.3	16.7	5.93	8.38
Lack of control over use	12.8	10.7	5.49	4.9	12.8	12	5.49	8.21
Relief from withdrawal	18	14.42	4.77	5.27	18.12	19	4.95	9.63

**Table 5:** Results of MANCOVA for HCQ subscales in the experimental and control groups

Variable	F	Sig.	Eta squared
Anticipation of positive outcome	9.32	.009**	.41
Intention and plan to use	.09	.77	.0
Desire to use	10.48	.006**	.45
Lack of control over use	.5	.49	.04
Relief from withdrawal	5.97	.03*	.32

DF = (1,19)

*DF* degrees of freedom

* *p* < .05

## Discussion

The purpose of the present study was to explore if neurofeedback training could enhance existing treatment for opiate dependence disorder. Although previous attempts at using neurofeedback as a treatment method have showed positive results, such studies have typically possessed a number of technical limitations that have reduced their usefulness especially as regards opiate disorders. For instance most of them have been focused on alcoholic patients and there are few experimental studies with a control group for opiate dependence disorders. Further, most of the latest research has consisted of case studies. Furthermore none of them has carried out studies comparing neurofeedback and Methadone or Buprenorphine maintenance treatment. Therefore in the present experimental study we examined the effectiveness of neurofeedback in comparison with MMT/BMT in two groups of opiate dependence patients, with a pre versus post treatment evaluation. This study focused on general psychological health and opiate craving in patients.

Neurofeedback was shown to decrease the craving to use substance and improve general mental health in opiate dependence patients. Some studies on alcohol dependence patients (Passini et al. 1977; Bodehnamer and Callaway 2004; Burkett et al. 2004; Raymond et al. 2005) found improvements like ours when comparing treatment to controls. For example, Scott et al. (2005) showed an increase in psychological health in mixed substance dependence patients receiving neurofeedback training, while Passini et al. (1977) and Peniston and Kulkosky (1989, 1991) found significant differences regarding anxiety signs in their study, that we could not achieve it in this study. Results obtained from the latest studies were based on long term neurofeedback training; however the current study has been able to obtain the same results, except for anxiety, but over a much shorter period of time. Continuing therapy could potentially lead to additional positive outcomes such as improvement in anxiety.

Our results, combined with those of others, suggest that neurofeedback training over a long period may be more effective than pharmacotherapy alone in treating substance use and in promoting mental health. Although pharmacotherapy can lead to some improvements in patients, side effects, instability, and the high risk of relapse, are some of the main limitations of using pharmacotherapy alone (Fagan 1994; Gossop et al. 2002). Neurofeedback attempts to address the fundamental operational functions of the brain and acts as a mechanism for the brain to self-regulate. Its goal is to correct irregular brain functions and consequently improve psychological abnormalities. Furthermore research confirms the stability of neurofeedback effects and its prevention of negative side effects (Hammond 2005). Thus pharmacotherapy can be used to maintain the initial balance between physiological and psychological health in substance dependent patients (Gossop et al. 2002), and then neurofeedback training can be used to guide the patient towards longer lasting health and balance.

There are several opinions about the fundamental mechanisms of effectiveness of neurofeedback training as a therapeutic method in substance use disorders. Several (Ochs 1992; Peniston 1994) suggest that the most active (and apparently transformational) properties of neurofeedback protocols in substance dependency treatment involve teaching participants to intentionally increase the amplitude and coherent interaction of both their alpha and theta brainwave frequencies in either of the brain locations. The mechanism of alpha-theta neurofeedback may lie in its ability to allow participants to better tolerate stress, anxiety, and anxiety eliciting situations, which are particularly evident during the initial phases of recovery (Scott et al. 2005).

Some other theories focus on conditional normalization of reinforcement systems in the brain. Blum et al. (2000), focusing on the reward deficiency syndrome that leads to substance craving, suggested that neurofeedback training can initiate a neurological normalizing shift. Following this idea, some studies stated that an apparent neurological “normalization” is responsible for shifting the trained subject into a physical state of comfortable calmness. When chemically dependent patients are calm they often have a neurologically based inability to experience pleasant feelings from simple stimulation (Fahrion et al. 1992; Salansky et al. 1998). Dysfunction of this pleasant feeling is the most important factor “forcing” patients to feel craving and to use substances (Kreek et al. 2005).

On the other hand Cowan (1994) suggested that the apparent effectiveness of such training may be due to the enhanced imprinting of positive temperance suggestions and the feeling of inner empowerment which the alpha-theta state seems to encourage. In another opinion, McPeak et al. (1991) suggested that self-induced altered-states such as those found in various forms of meditation, can sometimes replace the self destructive pursuit of alcohol and drugs. On the basis of this, Rosenfeld (1992) questioned whether there would be any difference between Peniston’s neurofeedback protocol, general relaxation, and hypnotic suggestion. Others suggest that the same results can be accomplished with meditation procedures alone (Taub et al. 1994).

Finally, as studies have shown, in the treatment of substance dependence disorder, no single program can lead to a cure by itself (Gossop et al. 2002). While taking into consideration the complexity of the dimensions of this disorder, treatment programs must be able to affect various factors and not be prone to the problems of previous methods, such as relapsing, instability, and other side effects (Trudeau 2000). The results of this study suggest neurofeedback training may produce additional benefits for increasing mental health in patients addicted to opiates, as well as being feasibly integrated with other methods.

In the current study, although we tried to control different factors in the process of neurofeedback training, due to the fact that we used technology in neurofeedback, and it is a new method, patient’s hope and motivation for the new treatment, could have heightened the effects noted. Despite this, the use of a placebo group could have strengthened the current design of the program and created more control over other aspects of the program. The high costs of the technology and time involved in neurofeedback, it was not feasible to use a placebo group. Future research projects need to consider incorporating attention/placebo condition to control the effects of interfering factors, so that the benefits of neurofeedback training can be seen more clearly. Inclusion of larger samples and longer term outcomes are needed to increase the level of validity of the results as well. Furthermore, the current research could not be carried out on patients with opiate dependence without also using the pharmacotherapy. In the future studies, one group should receive neurofeedback without receiving pharmacotherapy, which will permit a test of the individual and unique contributions of each approach. Finally in this study we did not repeat blood and urine tests for substance use. So we could not mention the abstinence range during the study. Future studies should include continuous assessments of these types.
